# Bacterial Contamination and Antimicrobial Resistance in Used Eye Cosmetic Products

**DOI:** 10.3390/microorganisms14051011

**Published:** 2026-04-30

**Authors:** Asta Aleksandravičienė, Giedrė Jarienė, Jolita Kirvaitienė, Rasa Volskienė, Kristina Dambrauskienė, Žaneta Maželienė

**Affiliations:** 1 Faculty of Medicine, Kauno Kolegija Higher Education Institution, Pramonės pr. 20, LT-50468 Kaunas, Lithuania; giedre.jariene@go.kauko.lt (G.J.); jolita.kirvaitiene@go.kauko.lt (J.K.); rasa.volskiene@go.kauko.lt (R.V.); zaneta.mazeliene@go.kauko.lt (Ž.M.); 2 Faculty of Medicine, Department of Psychiatry, Lithuanian University of Health Sciences, Eivenių St. 2, LT-50161 Kaunas, Lithuania; kristina.dambrauskiene@lsmu.lt; 3 Faculty of Veterinary Medicine, Institute of Microbiology and Virology, Lithuanian University of Health Sciences, A. Mickevičiaus g. 9, LT-44307 Kaunas, Lithuania

**Keywords:** eye cosmetic products, bacterial contamination, antimicrobial resistance, skin microbiome, coagulase-negative staphylococci, *Bacillus* spp., cosmetic microbiology, microbial safety

## Abstract

Eye cosmetic products are widely used and applied in close proximity to the ocular surface, making their microbiological safety particularly important. The aim of this study was to assess bacterial contamination in used eye cosmetic products, characterize the antimicrobial resistance profiles of the isolated bacteria, and perform molecular genotypic analysis. A total of 71 samples, including mascara, eyeliner, and eyeshadow, were analyzed. Microbiological analysis revealed that *Bacillus* spp. and coagulase-negative staphylococci (CoNS) were the predominant microorganisms, while no major pathogens such as *Staphylococcus aureus*, *Escherichia coli*, or *Pseudomonas aeruginosa* were detected. Antimicrobial susceptibility testing demonstrated high susceptibility of isolates to gentamicin, vancomycin, and linezolid, whereas resistance to benzylpenicillin and clindamycin was observed among *Staphylococcus* spp. Molecular identification based on 16S rRNA gene sequencing confirmed the presence of *Bacillus licheniformis*, *Bacillus subtilis*, *Staphylococcus epidermidis*, and *Staphylococcus warneri*, with sequences showing high similarity to globally distributed strains. Although the detected microorganisms were predominantly opportunistic, their presence in products applied near the eyes suggests a potential risk of microbial transfer to the ocular surface. These findings highlight the importance of proper hygiene practices, regular product replacement, and effective quality control measures to minimize microbial contamination and associated health risks.

## 1. Introduction

Eye cosmetic products, including mascara, eyeshadows, eyeliners, and eye pencils, are widely used and are applied in proximity to the ocular surface and periocular skin [1,2]. Because the eye is a highly sensitive anatomical structure, even minor microbial exposure may lead to irritation, inflammation, or infection. Cosmetic products applied near the ocular surface are therefore subject to stricter microbiological safety requirements, as the presence of pathogenic microorganisms such as *Staphylococcus aureus* and *Pseudomonas aeruginosa* may pose a significant risk of infection [3,4]. Consequently, microbiological safety of eye cosmetics is particularly important for maintaining ocular health and preventing adverse effects from contaminated products [5]. Cosmetic products are subject to regulatory frameworks designed to ensure their safety for consumer use. According to EU Regulation (EC) No. 1223/2009, these products are intended for application to external parts of the body and must comply with strict safety and quality requirements. In this context, Good Manufacturing Practices (GMP) play a crucial role in minimizing risks during production, including microbial contamination, and in ensuring compliance with established microbiological quality standards [6,7].

Microbial contamination may occur not only during manufacturing but also during storage, distribution, and subsequent consumer use. Repeated contact with the skin, eyelashes, and cosmetic applicators may introduce microorganisms into the product. These microorganisms may persist or proliferate, particularly when preservative systems gradually lose effectiveness during prolonged storage or environmental exposure [8,9]. Furthermore, once products are opened, maintaining microbiological quality largely depends on consumer practices, and improper handling may transform cosmetic products into potential reservoirs and vectors for microbial transmission [6,10]. Consumer practices such as prolonged use beyond the recommended shelf life, sharing of cosmetic products, improper storage, or inadequate hygiene may facilitate microbial transfer and multiplication in cosmetic formulations [6,10,11]. As a result, contaminated eye cosmetics may become a potential source of ocular irritation, allergic reactions, conjunctivitis, or more severe infections. These risks may be particularly significant among contact lens users or individuals with pre-existing ocular conditions [12,13].

Investigations of consumer-used cosmetic products have reported the presence of diverse microbial contaminants in eye cosmetic products, with microorganisms such as *S. aureus*, *Staphylococcus epidermidis*, and *P. aeruginosa* commonly identified in contaminated samples [3,6,14]. Similarly, studies examining cosmetic products used in beauty salons have demonstrated considerable levels of microbial contamination, suggesting that shared cosmetic products may serve as vehicles for microbial transmission between users [10]. Overall, these findings indicate that microbial contamination remains one of the most significant safety concerns associated with cosmetic products worldwide [6].

Among the microorganisms reported in contaminated cosmetic products, bacterial genera such as *Bacillus*, *Staphylococcus*, and *Pseudomonas* are most frequently identified and represent potential risk factors for ocular infections [3]. Species belonging to the genus Bacillus are capable of forming resistant spores that enable survival under unfavorable environmental conditions and in preservative-containing formulations [6,15]. Members of the genus *Staphylococcus*, including *S. aureus* and *S. epidermidis*, are commonly associated with the human skin microbiota. However, they may act as opportunistic pathogens and cause ocular infections such as conjunctivitis, keratitis, corneal ulcers, and endophthalmitis. Infections affecting the cornea or intraocular structures are of particular concern, as they may lead to severe complications and irreversible vision loss [13,16,17]. Furthermore, *P. aeruginosa* represents an important opportunistic pathogen frequently associated with severe ocular infections, especially among contact lens users [12,13].

Moreover, the growing prevalence of antimicrobial-resistant bacteria further amplifies concerns related to microbial contamination of cosmetic products. Antibiotic-resistant strains, including methicillin-resistant *S. aureus* (MRSA) and multidrug-resistant *P. aeruginosa*, have been increasingly reported worldwide and represent a major public health challenge [6,18]. The presence of antimicrobial-resistant microorganisms in cosmetic products applied near the ocular surface may complicate the treatment of infections and contribute to the dissemination of resistance determinants. This is particularly relevant in the context of cosmetic products, which may serve as reservoirs and transmission sources for resistant microorganisms during routine use [6,19]. Molecular characterization of microbial isolates provides important insights into pathogen identification, virulence factors, and resistance mechanisms, thereby improving our understanding of the potential risks associated with contaminated cosmetic products [20].

Although numerous studies have investigated microbial contamination of cosmetic products in different countries, data regarding the microbiological safety of eye cosmetic products used by consumers in Lithuania remain limited. Therefore, further investigation is necessary to evaluate the presence of potentially pathogenic and antibiotic-resistant microorganisms in these products. Taken together, these findings highlight the importance of assessing microbial contamination and antimicrobial resistance in eye cosmetic products, particularly those used by consumers under real-life conditions.

Data on microbial contamination of eye cosmetic products used under real consumer conditions in Lithuania are currently limited. Therefore, this study examines used eye cosmetic products collected from consumers in Lithuania.

The aim of this study was to assess bacterial contamination in used eye cosmetic products, characterize the antimicrobial resistance profiles of the isolated bacteria, and perform molecular genotypic analysis.

## 2. Materials and Methods

### 2.1. Sample Collection

Used eye cosmetic products were collected from volunteer consumers during the study period. The sample size was determined based on product availability and participant willingness to return used products, reflecting real-life consumer use conditions. A total of 71 used eye cosmetic products were collected. The sample set included 30 mascaras, 11 eyeliners, and 30 eyeshadows. At the time of sampling, all products were within their stated shelf life (not exceeding two years). As for controls, one new and unused product from each category was included. Each sample was assigned a unique identification code. Samples were collected aseptically using sterile cotton swabs. For mascara and eyeliner products, swabs were repeatedly inserted into the containers to obtain representative material. For eyeshadows, swabs were gently rubbed across the surface of the palettes.

### 2.2. Bacterial Cultivation, Isolation and Identification

Microbial recovery was performed using an enrichment-based approach. Enrichment media were prepared according to the manufacturer’s instructions, and Eugon broth (Liofilchem, Roseto degli Abruzzi, Italy) was used as the primary enrichment medium. Each sterile test tube contained 9 mL of broth, and approximately 1 g of sample (or an equivalent amount collected using sterile swabs) was introduced. Samples were incubated at 37 °C for 48 h.

In total, 74 enrichment cultures were prepared, including 71 cosmetic samples, three from unused control products, and one sterile control to verify medium sterility. Following enrichment, samples were streaked onto selective culture media for isolation of specific microorganisms. Mannitol Salt Agar (MSA), Cetrimide Agar, Bacillus cereus Agar, MacConkey Agar, and Sabouraud Dextrose Agar (all obtained from Liofilchem, Roseto degli Abruzzi, Italy) were used for the isolation of *Staphylococcus* spp., *P. aeruginosa*, *Bacillus* spp., Gram-negative bacteria including *Escherichia coli*, and yeasts and molds, respectively, following the manufacturer’s instructions. Plates were incubated at 37 °C for 48 h. Microbial growth was evaluated based on colony morphology, pigmentation, surface characteristics, and growth on selective media. Isolates were further characterized using microscopic and biochemical methods.

Gram staining was performed for all isolates, followed by microscopic examination under oil immersion. Presumptive *Staphylococcus* isolates were further identified using mannitol fermentation, coagulase (BioLife Solutions, Bothell, WA, USA), and DNase (Liofilchem, Roseto degli Abruzzi, Italy) tests, according to the manufacturers’ instructions. Mannitol fermentation was assessed by observing color change on MSA. Coagulase activity was determined using citrated rabbit plasma after incubation at 37 °C for 12 h. DNase activity was evaluated on DNase agar after 24 h incubation, with clear zones indicating positive results. Isolates positive for both coagulase and DNase were considered presumptive *Staphylococcus aureus*, whereas isolates negative for these tests were classified as coagulase-negative staphylococci (CoNS). Isolates grown on *Bacillus cereus* agar were identified as *Bacillus* spp. based on Gram-positive rod morphology observed by microscopy. CFU/g enumeration was outside the scope of this qualitative detection/identification study and was therefore not conducted.

Replication was performed at the level of independent samples (*n* = 71), with each sample processed using a standardized procedure.

### 2.3. Antimicrobial Susceptibility Testing

Antimicrobial susceptibility testing (AST) was performed using the Kirby–Bauer disk diffusion method according to the 2025 guidelines of the European Committee on Antimicrobial Susceptibility Testing (EUCAST). Bacterial suspensions were adjusted to 0.5 McFarland turbidity and inoculated onto Mueller–Hinton agar plates. The following antibiotic discs (Liofilchem, Roseto degli Abruzzi, Italy) were employed: β-lactams (benzylpenicillin, ampicillin, cefoxitin, cefazolin), aminoglycosides (gentamicin), macrolides (erythromycin), lincosamides (clindamycin), glycopeptides (vancomycin), oxazolidinones (linezolid), and folate pathway inhibitors (trimethoprim/sulfamethoxazole). Plates were incubated at 35–37 °C for 18–24 h, and inhibition zones were measured in millimeters. Results were interpreted as susceptible, intermediate, or resistant according to the latest EUCAST clinical breakpoints. Quality control strains used in antimicrobial susceptibility testing were *Staphylococcus epidermidis* ATCC 12228 and *Bacillus subtilis* ATCC 6633.

### 2.4. DNA Extraction and PCR Amplification

Genomic DNA was extracted from bacterial isolates using the PureLink Genomic DNA Mini Kit (Thermo Fisher Scientific, Vilnius, Lithuania) according to the manufacturer’s instructions. Bacterial identification was performed by amplification of approximately 1500 bp of the 16S rRNA gene using universal primers 27F (5′-AGAGTTTGATCCTGGCTCAG-3′) and 1541R (5′-AAGGAGGTGATCCAGCCGCA-3′) [21,22]. PCR reactions were carried out in a total volume of 25 µL containing 1xDreamTaq Green PCR Master Mix (Thermo Fisher Scientific, Vilnius, Lithuania), 0.4 pmol of each primer, and 50 ng of template DNA. Thermal cycling conditions included an initial denaturation at 94 °C for 3 min, followed by 30 cycles of denaturation at 94 °C for 45 s, annealing at 55 °C for 30 s, and extension at 72 °C for 2 min, with a final extension at 72 °C for 7 min. PCR products were separated by electrophoresis on 1.5% agarose gel containing ethidium bromide and visualized under UV illumination. Positive and negative controls were included in each PCR run.

### 2.5. DNA Sequencing and Phylogenetic Analysis

Representative PCR products were purified using the GeneJET Gel Extraction Kit (Thermo Fisher Scientific, Vilnius, Lithuania) and sequenced at the Nanodiagnostika Center (Vilnius, Lithuania). Obtained sequences were edited using MEGA X software (version 10.2.6) [23] and compared with reference sequences available in the GenBank database using the NCBI BLAST tool (https://blast.ncbi.nlm.nih.gov/Blast.cgi, accessed on 23 April 2026).

### 2.6. GenBank Accession Numbers

Representative sequences obtained in this study were deposited in the GenBank database under the following accession numbers: *Staphylococcus epidermidis* (PX946876, PX946877, PX946878), *Staphylococcus warneri* (PX947488), *Bacillus licheniformis* (PZ152083, PZ152084, PZ152085), and *Bacillus subtilis* (PZ152086). Representative isolates were selected for 16S rRNA gene sequencing based on product category and bacterial group. As identical sequences were obtained, representative sequences were deposited in GenBank.

### 2.7. Statistical Analysis

Microbial prevalence was expressed as proportions (n/N) with 95% confidence intervals (CI) calculated using the Wilson method. Differences between groups were analyzed using Fisher’s exact test, and effect sizes were expressed as risk ratios (RR) with 95% CI. For product type stratified AST comparisons (mascara vs. eyeliner vs. eyeshadow), we used two-sided Fisher’s exact tests (pairwise) for resistance (R), and we additionally performed a sensitivity analysis using non-susceptibility (NS = I + R). Because several comparisons involved small cell counts, we used Fisher’s exact test (two-sided) for categorical comparisons. Alongside *p*-values, we report effect sizes (risk ratios or odds ratios) with 95% confidence intervals to reflect the magnitude and uncertainty of effects. Where applicable, we note that non-significant results may reflect limited statistical power due to the sample size. For antimicrobial susceptibility testing, exact Clopper–Pearson confidence intervals were calculated. For zero-event observations, upper confidence limits were estimated using the rule of three (3/n). Statistical analyses were performed using IBM SPSS Statistics version 29.0.

## 3. Results

### 3.1. Frequency of Microbial Isolation

A total of 71 used eye cosmetic products collected from individual consumers were analyzed, including eyeliner/pencils (n = 11), mascara (n = 30), and eyeshadow (n = 30). The most frequently detected microorganisms were *Bacillus* spp. and coagulase-negative staphylococci (CoNS). *Bacillus* spp. were identified in 72.7% (95% CI: 43.4–90.3) of eyeliner/pencil samples, 60.0% (95% CI: 42.3–75.4) of mascara samples, and 66.7% (95% CI: 48.8–80.8) of eyeshadow samples. CoNS were detected in 63.6% (95% CI: 35.4–84.8), 73.3% (95% CI: 55.6–85.8), and 53.3% (95% CI: 36.1–69.8) of samples, respectively. No samples yielded *S. aureus*, *E. coli*, fungi, or *P. aeruginosa*. Negative control samples (n = 3) showed no microbial growth. Overall, *Bacillus* spp. were detected in 46/71 products (64.8%; 95% CI: 53.2–74.9) and coagulase-negative staphylococci (CoNS) in 45/71 products (63.4%; 95% CI: 51.8–73.6). For transparency, we report detection rates as n/N (%) across product types. Detailed results are presented in Table 1.

### 3.2. Comparison Between Product Types

Using Fisher’s exact test, detection rates did not differ significantly between product types for either *Bacillus* spp. or CoNS (all *p* > 0.05), and effect sizes are reported as RR with 95% CIs. Pairwise comparisons of microbial detection rates between product categories revealed no statistically significant differences. For *Bacillus* spp., the risk ratio (RR) was 1.21 (95% CI: 0.76–1.93; *p* = 0.716) for eyeliner/pencil versus mascara, 1.09 (95% CI: 0.70–1.70; *p* > 0.999) for eyeliner/pencil versus eyeshadow, and 0.90 (95% CI: 0.61–1.32; *p* = 0.789) for mascara versus eyeshadow. For CoNS, the RR was 0.87 (95% CI: 0.53–1.43; *p* = 0.701), 1.19 (95% CI: 0.68–2.09; *p* = 0.726), and 1.38 (95% CI: 0.92–2.05; *p* = 0.180), respectively. All confidence intervals included 1, indicating that microbial detection rates did not differ significantly between product types. No cases of *S. aureus*, *E. coli*, fungi, or *P. aeruginosa* were detected. Based on the rule of three (3/n), the upper 95% confidence interval limits were estimated as 27.3% for eyeliner/pencil samples, 10.0% for mascara and eyeshadow samples, and 4.2% overall, indicating a low probability of occurrence within the analyzed population.

### 3.3. Antimicrobial Susceptibility of Staphylococcus spp.

Antimicrobial susceptibility testing of 45 *Staphylococcus* spp. isolates revealed variable resistance patterns (Table 2). Overall, 30/45 (66.7%) staphylococcal isolates were resistant to at least one tested anti-biotic, and 42/45 (93.3%) showed non-susceptibility (I or R) to at least one agent. The highest susceptibility rates were observed for gentamicin, linezolid, and vancomycin, with 100% of isolates classified as susceptible. Trimethoprim/sulfamethoxazole also demonstrated high efficacy (97.8% susceptible). Cefoxitin showed 95.6% susceptibility, indicating a low prevalence of methicillin-resistant isolates. In contrast, resistance to β-lactam antibiotics was more pronounced. Benzylpenicillin showed 40.0% resistance, while ampicillin exhibited 8.9% resistance and a high proportion of intermediate susceptibility (57.8%). Moderate resistance was observed for erythromycin (13.3%), with 46.7% intermediate susceptibility. Cefazolin showed 15.6% resistance, while clindamycin demonstrated 26.7% resistance. Overall, these findings indicate that gentamicin and trimethoprim/sulfamethoxazole remain highly effective, whereas resistance to benzylpenicillin and clindamycin should be considered when selecting empirical therapy.

Differences in antimicrobial resistance patterns across product types (mascara, eyeliner, eyeshadow) were assessed using two-sided Fisher’s exact tests. No statistically significant differences were observed for any antibiotic (all *p* > 0.05). Detailed product type-stratified resistance frequencies and corresponding *p* values are provided in Table 3. A sensitivity analysis using non-susceptibility (NS = I + R) yielded consistent results (all *p* > 0.05). Limited statistical power should be considered for the smallest group (eyeliner, n = 7).

### 3.4. Antimicrobial Susceptibility of Bacillus spp.

Antimicrobial susceptibility testing of 46 *Bacillus* spp. isolates demonstrated varying resistance patterns (Table 4). The highest resistance rate was observed for clindamycin (58.7%), followed by erythromycin (26.1%). Intermediate susceptibility was most frequently observed for ciprofloxacin (15.2%). All isolates were fully susceptible to norfloxacin (100%), as well as to vancomycin and linezolid (100%). These results indicate that norfloxacin, vancomycin, and linezolid remain highly effective against *Bacillus* spp., whereas clindamycin and erythromycin show considerable resistance.

Differences in antimicrobial resistance patterns across product types (mascara, eyeliner/pencil, eyeshadow) among *Bacillus* spp. isolates were assessed using two-sided Fisher’s exact tests. In contrast to *Staphylococcus* spp., significant differences between product types were observed for selected antibiotics. Clindamycin resistance differed significantly between mascara and eyeliner (*p* = 0.007) and between eyeliner and eyeshadow (*p* = 0.035), while erythromycin resistance also varied by product type (*p* = 0.022 for mascara vs. eyeliner; *p* = 0.042 for mascara vs. eyeshadow). Ciprofloxacin resistance was observed predominantly among eyeshadow isolates, with a significant difference between mascara and eyeshadow (*p* = 0.024). No resistance to vancomycin, linezolid, or norfloxacin was detected across any product type (all R = 0; *p* = 1.000). Detailed product type-stratified resistance frequencies (n/N, %) and exact-test *p* values are provided in Table 5. Limited statistical power should be considered for the smallest group (eyeliner/pencil; n = 8 in the *Bacillus* dataset).

### 3.5. Molecular Identification and Phylogenetic Analysis

**Figure 1:**
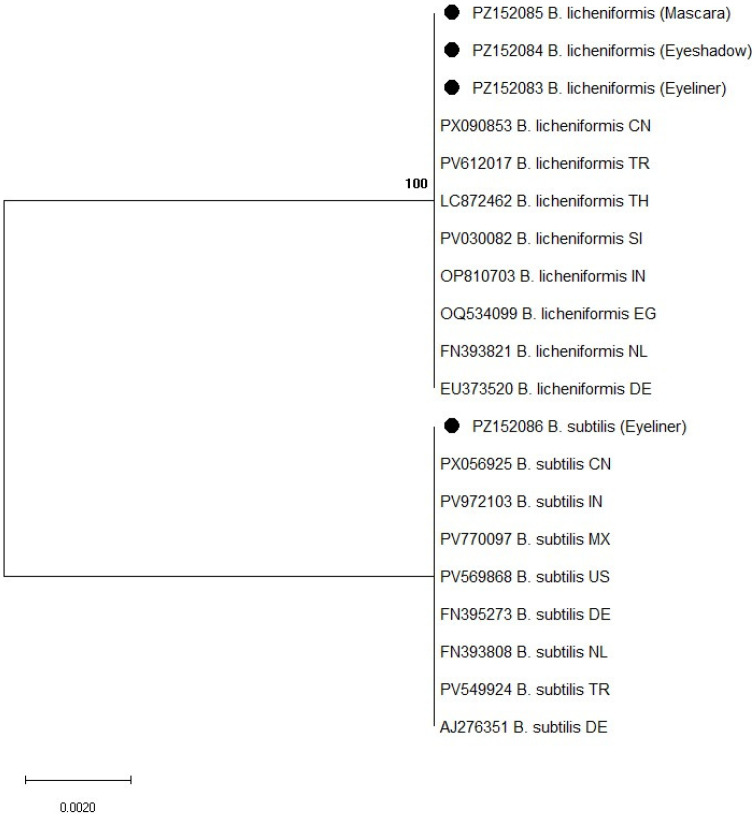
Phylogenetic tree of *Bacillus* spp. based on 16S rRNA gene sequences constructed using the Maximum Likelihood method and Tamura–3 parameter model. Bootstrap values are shown at branch nodes. Reference sequences were retrieved from the GenBank database. Accession numbers are followed by species name and country of origin, indicated using ISO two-letter country codes. Isolates obtained in this study are marked with black circles. Abbreviations: CN—China; TR—Turkey; TH—Thailand; SI—Slovenia; IN—India; EG—Egypt; NL—Netherlands; DE—Germany; MX—Mexico; US—United States.

**Figure 2:**
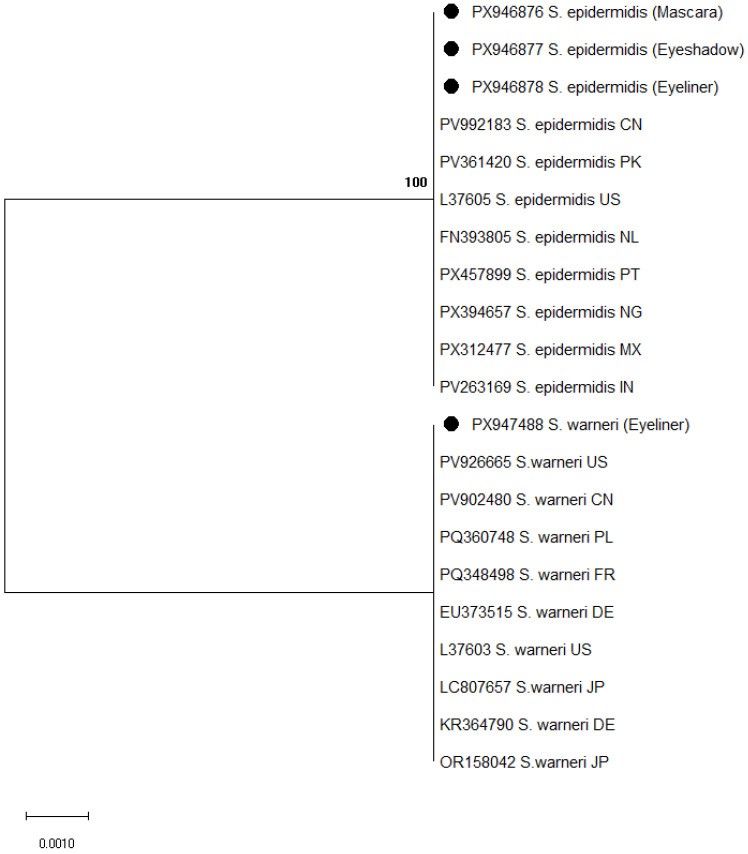
Phylogenetic tree of *Staphylococcus* spp. based on 16S rRNA gene sequences constructed using the Maximum Likelihood method and Tamura–3 parameter model. Bootstrap values are shown at branch nodes. Reference sequences were retrieved from the GenBank database. Accession numbers are followed by species name and country of origin, indicated using ISO two-letter country codes. Isolates obtained in this study are marked with black circles. Abbreviations: CN—China; PK—Pakistan; US—United States; NL—Netherlands; PT—Portugal; NG—Nigeria; MX—Mexico; IN—India; FR—France; DE—Germany; JP—Japan; PL—Poland.

Partial 16S rRNA gene sequencing was used for the molecular identification of bacterial isolates recovered from mascara, eyeshadow, and eyeliner samples. A total of 91 bacterial isolates were subjected to 16S rRNA gene sequencing, including 15 isolates from eyeliner (8 *Bacillus* spp. and 7 coagulase-negative staphylococci), 40 from mascara (18 *Bacillus* spp. and 22 coagulase-negative staphylococci), and 36 from eyeshadow samples (20 *Bacillus* spp. and 16 coagulase-negative staphylococci). Sequence analysis confirmed the presence of species belonging to the genera *Bacillus* and *Staphylococcus*, supporting phenotypic identification results. Phylogenetic analysis further confirmed the taxonomic assignment of the isolates, as shown in Figure 1 and Figure 2.

Among *Bacillus* species, *Bacillus licheniformis* was the most frequently detected and was identified in all three product categories. The obtained sequences showed 100% similarity to GenBank reference sequences, including strains from Europe (Germany: EU373520; the Netherlands: EU008514) and other regions (China: PX006853; Nigeria: OP703543), indicating high genetic conservation. In the phylogenetic tree (Figure 1), these isolates clustered closely with corresponding reference sequences from different geographical regions.

*Bacillus subtilis* was detected exclusively in eyeliner samples. These isolates also exhibited 100% similarity to reference strains from Germany (EU373519), the Netherlands (FN393811), and the United States (PV596888), and grouped within the same clade as reference sequences in the phylogenetic analysis (Figure 1).

Among *Staphylococcus* species, *S. epidermidis* was detected in all product categories and showed 100% similarity to reference sequences from the Netherlands (FN393805), China (PV992183), and the United States (L37605). In the phylogenetic tree (Figure 2), these isolates clustered with globally distributed reference strains. *Staphylococcus warneri* was identified only in eyeliner samples and exhibited 100% similarity to sequences from Germany (KR364790), France (PQ348498), and Japan (LC807657), forming a distinct cluster with corresponding reference sequences (Figure 2).

Overall, the genetic analysis demonstrated that the identified bacterial isolates are highly conserved and closely related to globally distributed environmental and skin-associated microorganisms.

## 4. Discussion

The present study demonstrated that decorative eye cosmetic products may harbor microorganisms commonly associated with environmental sources and human skin microbiota. Microbiological analysis revealed that *Bacillus* spp. and coagulase-negative staphylococci (CoNS) were the predominant microorganisms isolated from mascara, eyeliner, and eyeshadow products. In this study, the absence of pathogens such as *E. coli*, *P. aeruginosa*, and fungi indicates that the tested products were free from major pathogenic contaminants at the time of analysis. This finding is relevant because PN-EN ISO 17516 [24] defines specified microorganisms for cosmetic microbiological control (including *Candida albicans*, *E. coli*, *P. aeruginosa* and *S. aureus*); however, no detection in a finite sample should not be interpreted as evidence of zero prevalence [25]. However, the presence of opportunistic microorganisms suggests that eye cosmetics may still represent potential reservoirs for microbial transfer to the ocular surface.

The bacterial contamination observed in this study is consistent with previous investigations demonstrating that cosmetic products applied near the eyes, particularly mascaras and eyeliners, are susceptible to microbial colonization due to repeated contact with eyelashes, skin, and environmental surfaces. Bashir et al. [3] reported that more than half of the tested cosmetic products contained bacterial contaminants, with *Bacillus* spp. and Staphylococcus spp. among the most frequently isolated microorganisms. Among the identified species, coagulase-negative *Staphylococcus* species such as *Staphylococcus hominis*, *Staphylococcus haemolyticus*, and *Staphylococcus capitis* were commonly detected in eye cosmetic products.

Similarly, Ghias and Fozouni [10] demonstrated high levels of microbial contamination in cosmetic products used in beauty salons, including eye makeup products such as mascaras, eyeliners, and eyeshadows. In their study, microbial contamination was detected in 56.25% of all analyzed samples, with *S. aureus* and *C. albicans* identified as the predominant bacterial and fungal pathogens, respectively. Although eye cosmetic products exhibited lower contamination rates (approximately 20%) compared to other product categories, the presence of these potentially pathogenic microorganisms highlights the risk associated with repeated use and shared application tools. Comparable findings were reported by Dadashi and Dehghanzadeh [26], who identified bacterial and fungal contamination in shared cosmetic kits used in beauty salons, including eye cosmetic products, with microorganisms such as *S. aureus*, *P. aeruginosa*, and *Candida* spp. detected. In addition, Almukainzi et al. [27] demonstrated that commonly used topical cosmetic products frequently harbor microbial contaminants, including both Gram-positive and Gram-negative bacteria, emphasizing the ongoing concerns regarding cosmetic product safety. A recent review by da Silva et al. [6] further confirmed that microbial contamination remains one of the most common quality concerns in cosmetic products worldwide, particularly in products applied directly to the face and periocular area.

Among the microorganisms detected in this study, *Bacillus* species represented the most prevalent bacterial group. Molecular identification confirmed that *B. licheniformis* was the most frequently detected species and was present in all three categories of cosmetic products.

The predominance of *Bacillus* spp. in cosmetic products has been reported in several previous studies [3,6,15]. This can be attributed to their ability to form endospores, which enable survival under adverse environmental conditions, including exposure to preservatives and fluctuating storage conditions. Spores can persist in cosmetic products for extended periods and may germinate when environmental conditions become favorable, this explains their frequent detection in microbiological analyses [6]. The high genetic similarity between the *B. licheniformis* isolates identified in this study and reference strains reported from diverse geographical regions indicates that these bacteria represent globally distributed environmental microorganisms associated with soil, dust, and human skin microbiota.

*Bacillus subtilis* was detected exclusively in eyeliner samples, suggesting potential product-specific contamination patterns. This observation may be related to differences in formulation, packaging, or frequency of direct contact with the skin. Previous studies have also reported the presence of *B. subtilis* in cosmetic products due to its ubiquitous environmental distribution and resistance to environmental stress [6,28]. Although generally considered non-pathogenic, *B. subtilis* may act as an opportunistic microorganism under certain conditions, particularly when introduced into sensitive anatomical sites such as the ocular surface, as has been reported for *Bacillus* species in cosmetic environments [29].

The second predominant group of microorganisms detected in this study consisted of coagulase-negative staphylococci. Molecular analysis identified *S. epidermidis* and *S. warneri*, both of which clustered closely with globally distributed reference strains, confirming the reliability of species identification. *S. epidermidis* was detected in mascara, eyeshadow, and eyeliner samples and is a well-known component of the normal human skin microbiota [17]. This species is frequently reported in cosmetic products, likely due to repeated contact between applicators and the user’s skin or eyelashes [11,14].

The complete sequence identity observed among *Bacillus* and *Staphylococcus* isolates suggests low genetic diversity within the analyzed bacterial populations. This may indicate that contamination originates from common sources, such as the human skin microbiota or environmental exposure during product use. The absence of sequence variability also suggests that cosmetic formulations may selectively favor the survival of specific resilient bacterial strains. Although the identified bacteria are widely distributed and not considered primary pathogens, their persistence in eye cosmetic products may facilitate repeated exposure to the ocular surface, potentially increasing the risk of opportunistic infections, particularly in individuals with compromised ocular defense mechanisms.

These observations are consistent with previous studies demonstrating that cosmetic products may become contaminated during storage and repeated consumer use, even when preservative systems are present, as continuous exposure to environmental microorganisms and repeated handling may facilitate microbial survival and proliferation within the product [30,31]. This phenomenon may be further explained by the variable efficacy of preservative systems, which can depend on product formulation, storage conditions, and duration of use, thereby allowing microorganisms to persist despite the presence of antimicrobial agents [32,33,34]. Brannan et al. [35] reported that in vitro preservation tests may underestimate contamination risk under real-use conditions. Similarly, Badyeh et al. [36] identified microbial contamination in commercially available cosmetic products, including potentially pathogenic bacteria such as *S. aureus* and *P. aeruginosa.* According to da Silva et al. [6], contamination by coagulase-negative staphylococci is strongly associated with consumer handling practices, including prolonged product use, sharing of cosmetic items, and inadequate hygiene of applicators.

*Staphylococcus warneri*, detected exclusively in eyeliner samples, has also been reported as a contaminant in cosmetic products [15]. Although traditionally considered a commensal organism, it has been increasingly recognized as an opportunistic pathogen capable of causing infections in immunocompromised individuals or when epithelial barriers are compromised [37]. In the context of eye cosmetics, repeated exposure to such microorganisms may contribute to bacterial transfer to the ocular surface, particularly among contact lens users or individuals with compromised ocular defense mechanisms [11].

Another important aspect evaluated in this study was antimicrobial susceptibility of isolated microorganisms. Coagulase-negative staphylococci demonstrated resistance to penicillin and clindamycin, whereas susceptibility to gentamicin, cefoxitin, and trimethoprim/sulfamethoxazole remained high. The observed resistance patterns are consistent with known staphylococcal resistance mechanisms. Resistance to β-lactams is commonly mediated by β-lactamase production and/or the mecA-encoded penicillin-binding protein (PBP2a), while cefoxitin-based testing is widely used to screen for methicillin resistance. Resistance to macrolide–lincosamide antibiotics may be associated with erm-mediated target site modification (23S rRNA methylation) and/or efflux mechanisms [38,39]. These findings are consistent with previous reports indicating that CoNS may exhibit resistance to β-lactam antibiotics, partly due to the presence of resistance genes and their ability to form biofilms [14,18]. Although the detected isolates did not represent highly resistant clinical pathogens, the presence of antibiotic-resistant bacteria in cosmetic products remains a concern, as these microorganisms may act as reservoirs and potential sources of antimicrobial resistance dissemination [6,19]. In antimicrobial resistance (AMR) surveillance, multidrug resistance (MDR) and extensive drug resistance (XDR) are defined based on isolate-level susceptibility profiles across antimicrobial categories. Reliable MDR/XDR classification and species–resistance association analysis require isolate-linked datasets [40]. Because the AMR results in this study were summarized at the antibiotic level, such analyses were not performed to avoid potential misclassification. This is recognized as a limitation and should be addressed in future studies. From a One Health perspective, consumer products repeatedly applied to the skin and periocular area may act as interfaces between humans and the environment, facilitating the circulation of commensal microorganisms and their resistance determinants. Coagulase-negative staphylococci (CoNS), although commonly considered commensals, are recognized as opportunistic pathogens and important reservoirs of transferable resistance genes. Therefore, the presence of resistant CoNS in eye cosmetic products may have clinical relevance and contribute to the broader community resistome [41,42,43]. These findings also highlight the importance of appropriate microbiological quality control and regulatory oversight of cosmetic products to minimize potential health risks.

The findings of the present study are consistent with those reported in European studies, where contamination is predominantly associated with coagulase-negative staphylococci and *Bacillus* spp., rather than highly pathogenic bacteria [3,6]. In contrast, studies conducted in other regions more frequently report the presence of major pathogens such as *S. aureus* and *P. aeruginosa* [10,26,27]. These differences may be attributed to variations in manufacturing standards, regulatory enforcement, hygiene practices, and consumer behavior across regions.

Overall, the results of this study demonstrate that decorative eye cosmetic products may harbor opportunistic microorganisms originating from environmental sources or the human skin microbiota. Although these microorganisms are not typically primary pathogens, their presence in products applied near the ocular surface represents a potential route of microbial exposure. Repeated use of contaminated cosmetic products may contribute to ocular irritation, conjunctivitis, keratitis, or secondary infections, particularly among individuals with compromised ocular defense mechanisms [44]. These observations emphasize the role of consumer use conditions in shaping microbial contamination patterns and underline the importance of proper product handling and hygiene practices.

These findings also have regulatory and risk management implications, particularly for cosmetics intended for use in the eye area. International and European microbiological quality guidelines emphasize product safety during intended use and highlight stricter requirements for higher-risk product categories [45]. Our real-use findings underscore the importance of robust preservation strategies, clear consumer guidance (e.g., hygiene practices and timely replacement), and post-market surveillance to minimize microbial exposure in the periocular area.

The absence of quantitative enumeration (CFU/g) limits the assessment of overall contamination levels and severity. In addition, detailed sampling metadata (e.g., product age and usage patterns) were not systematically collected, restricting the ability to evaluate factors associated with contamination. The relatively modest sample size may also limit the generalizability of the findings. Despite these constraints, the combined phenotypic (AST) and molecular characterization provides valuable insight into microbial contamination and antimicrobial resistance profiles in used eye cosmetic products.

## 5. Conclusions

This study demonstrates that used eye cosmetic products may harbor opportunistic microorganisms primarily originating from environmental sources and the human skin microbiota. *Bacillus* spp. and coagulase-negative staphylococci were identified as the predominant contaminants across all product types, whereas major pathogenic bacteria were not detected. Our results demonstrate the presence of antimicrobial-resistant staphylococci in used eye cosmetic products, suggesting a potential microbiological risk and the need for further investigation. These findings indicate that routine consumer use of eye cosmetic products may contribute to microbial exposure and emphasize the need for improved hygiene practices, consumer awareness, and effective manufacturing and preservation strategies to ensure product safety.

## Figures and Tables

**Table 1:** Microbial isolates detected in cosmetic products (*n* = 71).

Microorganism	Eyeliner/Pencil *n* (%) (n = 11)	Mascara *n* (%) (n = 30)	Eyeshadow *n* (%) (n = 30)	Negative Control *
*Bacillus* spp.	8 (72.7%)	18 (60.0%)	20 (66.7%)	0 (0)
Coagulase-negative staphylococci (CoNS)	7 (63.6%)	22 (73.3%)	16 (53.3%)	0 (0)
*Staphylococcus aureus*	0 (0)	0 (0)	0 (0)	0 (0)
*Escherichia coli*	0 (0)	0 (0)	0 (0)	0 (0)
Fungi	0 (0)	0 (0)	0 (0)	0 (0)
*Pseudomonas aeruginosa*	0 (0)	0 (0)	0 (0)	0 (0)

* Negative control: One new, unused product from each category (total n = 3). No microbial growth was detected.

**Table 2:** Antibiotic susceptibility of coagulase-negative staphylococci (CoNS) isolates (*n* = 45).

Class	Antibiotic	S n (%)	I n (%)	R n (%)
β-lactam	Benzylpenicillin	25 (55.6)	2 (4.4)	18 (40.0)
β-lactam	Ampicillin	15 (33.3)	26 (57.8)	4 (8.9)
β-lactam	Cefazolin	38 (84.4)	0 (0.0)	7 (15.6)
β-lactam	Cefoxitin (screen)	43 (95.6)	0 (0.0)	2 (4.4)
Macrolide	Erythromycin	18 (40.0)	21 (46.7)	6 (13.3)
Lincosamide	Clindamycin	33 (73.3)	0 (0.0)	12 (26.7)
Aminoglycoside	Gentamicin	45 (100.0)	0 (0.0)	0 (0.0)
Folate pathway inhibitors	Trimethoprim/sulfamethoxazole	44 (97.8)	0 (0.0)	1 (2.2)
Glycopeptide	Vancomycin	45 (100.0)	0 (0.0)	0 (0.0)
Oxazolidinone	Linezolid	45 (100.0)	0 (0.0)	0 (0.0)

Footnote: S—Susceptible (standard dosing regimen); I—Susceptible, increased exposure; R—Resistant.

**Table 3:** Antimicrobial resistance (R) by product type among coagulase-negative staphylococci isolates (*n* = 45).

Antibiotic	Mascara (T) (*n* = 22) R n/N (%)	Eyeliner (E) (*n* = 7) R n/N (%)	Eyeshadow (S) (*n* = 16) R n/N (%)	*p* (T vs. E)	*p* (T vs. S)	*p* (E vs. S)
Cefoxitin (screen)	1/22 (4.5)	1/7 (14.3)	0/16 (0.0)	0.431	1.000	0.304
Benzylpenicillin	10/22 (45.5)	1/7 (14.3)	8/16 (50.0)	0.202	1.000	0.176
Ampicillin	2/22 (9.1)	1/7 (14.3)	1/16 (6.2)	1.000	1.000	0.526
Erythromycin	2/22 (9.1)	1/7 (14.3)	3/16 (18.8)	1.000	0.632	1.000
Cefazolin	3/22 (13.6)	2/7 (28.6)	2/16 (12.5)	0.569	1.000	0.557
Trimethoprim/sulfamethoxazole	0/22 (0.0)	0/7 (0.0)	1/16 (6.2)	1.000	0.421	1.000
Gentamicin	0/22 (0.0)	0/7 (0.0)	0/16 (0.0)	1.000	1.000	1.000
Clindamycin	7/22 (31.8)	3/7 (42.9)	2/16 (12.5)	0.665	0.254	0.142
Linezolid	0/22 (0.0)	0/7 (0.0)	0/16 (0.0)	1.000	1.000	1.000

Footnote: Pairwise comparisons were assessed using two-sided Fisher’s exact test (*p*-values shown). S—Susceptible (standard dosing regimen); R—Resistant.

**Table 4:** Antibiotic susceptibility of *Bacillus* spp. isolates (*n* = 46).

Class	Antibiotic	S n (%)	I n (%)	R n (%)
Fluoroquinolone	Ciprofloxacin	32 (69.6)	7 (15.2)	7 (15.2)
Fluoroquinolone	Norfloxacin	46 (100.0)	0 (0.0)	0 (0.0)
Macrolide	Erythromycin	30 (65.2)	4 (8.7)	12 (26.1)
Lincosamide	Clindamycin	18 (39.1)	1 (2.2)	27 (58.7)
Glycopeptide	Vancomycin	46 (100.0)	0 (0.0)	0 (0.0)
Oxazolidinone	Linezolid	46 (100.0)	0 (0.0)	0 (0.0)

Footnote: S—Susceptible (standard dosing regimen); I—Susceptible, increased exposure; R—Resistant.

**Table 5:** Antimicrobial resistance (R) by product type among *Bacillus* spp. isolates (*n* = 46).

Antibiotic	Mascara (T) (*n* = 17) R n/N (%)	Eyeliner (E) (*n* = 8) R n/N (%)	Eyeshadow (S) (*n* = 21) R n/N (%)	*p* (T vs. E)	*p* (T vs. S)	*p* (E vs. S)
Ciprofloxacin	0/17 (0.0)	1/8 (12.5)	6/21 (28.6)	0.320	0.024	0.635
Vancomycin	0/17 (0.0)	0/8 (0.0)	0/21 (0.0)	1.000	1.000	1.000
Norfloxacin	0/17 (0.0)	0/8 (0.0)	0/21 (0.0)	1.000	1.000	1.000
Clindamycin	13/17 (76.5)	1/8 (12.5)	13/21 (61.9)	0.007	0.486	0.035
Linezolid	0/17 (0.0)	0/8 (0.0)	0/21 (0.0)	1.000	1.000	1.000
Erythromycin	9/17 (52.9)	0/8 (0.0)	4/21 (19.0)	0.022	0.042	0.552

Footnote: Pairwise comparisons were assessed using two-sided Fisher’s exact test (*p*-values shown). S—Susceptible (standard dosing regimen); R—Resistant.

## Data Availability

The original contributions presented in this study are included in the article. Further inquiries can be directed to the corresponding author.
